# 5-(3-Chloro­phen­yl)-2-phenyl-3,4-dihydro-2*H*-pyrrole

**DOI:** 10.1107/S160053681003847X

**Published:** 2010-10-09

**Authors:** Guiqiu Yang, Xiaoqing Su, Liang Lv

**Affiliations:** a Shenyang Universtity of Chemical Technology, Shenyang 110142, People’s Republic of China; b Agrochemicals Division, Shenyang Research Institute of Chemical Industry, Shenyang 110021, People’s Republic of China

## Abstract

In the title compound, C_16_H_14_ClN, the conformation of the five-membered ring approximates to an envelope with a C atom as the flap. The dihedral angle between the aromatic rings is 78.71 (9)°.

## Related literature

For chemical background to pyrrolines, see: Tsuge *et al.* (1987 ▶).
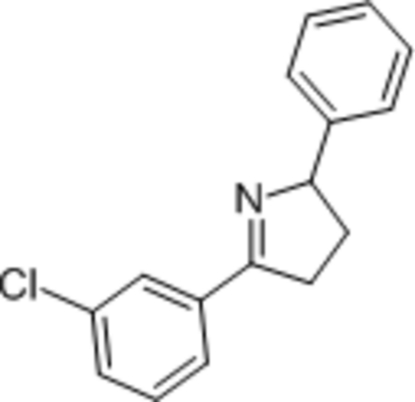


## Experimental

### 

#### Crystal data

C_16_H_14_ClN
*M* *_r_* = 255.73
Monoclinic, 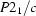

*a* = 18.2543 (18) Å
*b* = 5.6398 (5) Å
*c* = 13.0095 (13) Å
β = 97.129 (2)°
*V* = 1329.0 (2) Å^3^
*Z* = 4
Mo *K*α radiation
μ = 0.27 mm^−1^
*T* = 296 K
0.38 × 0.32 × 0.20 mm

#### Data collection

Bruker SMART CCD diffractometer
Absorption correction: multi-scan (*SADABS*; Bruker, 2001 ▶) *T*_min_ = 0.905, *T*_max_ = 0.948
6409 measured reflections
2344 independent reflections
1856 reflections with *I* > 2σ(*I*)
*R*_int_ = 0.017

#### Refinement

*R*[*F* ^2^ > 2σ(*F* ^2^)] = 0.033
*wR*(*F* ^2^) = 0.091
*S* = 1.06
2344 reflections
163 parameters
H-atom parameters constrained
Δρ_max_ = 0.13 e Å^−3^
Δρ_min_ = −0.18 e Å^−3^

### 

Data collection: *SMART* (Bruker, 2001 ▶); cell refinement: *SAINT* (Bruker, 2001 ▶); data reduction: *SAINT*; program(s) used to solve structure: *SHELXS97* (Sheldrick, 2008 ▶); program(s) used to refine structure: *SHELXL97* (Sheldrick, 2008 ▶); molecular graphics: *SHELXTL* (Sheldrick, 2008 ▶); software used to prepare material for publication: *SHELXTL*.

## Supplementary Material

Crystal structure: contains datablocks I, global. DOI: 10.1107/S160053681003847X/hb5651sup1.cif

Structure factors: contains datablocks I. DOI: 10.1107/S160053681003847X/hb5651Isup2.hkl

Additional supplementary materials:  crystallographic information; 3D view; checkCIF report

## Figures and Tables

**Fig. 1.**
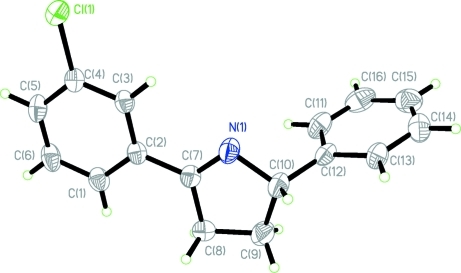
The molecular structure of (I), with 30% probability displacement ellipsoids.

**Fig. 2.**
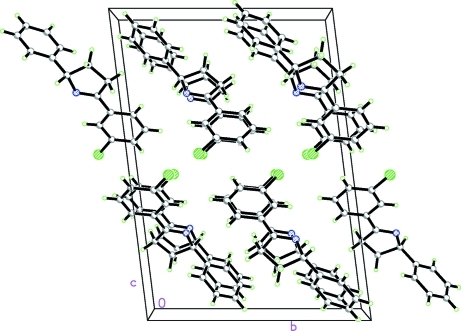
Crystal packing of (I).

## supplementary crystallographic information

### Experimental

To a 500 ml flask *N*-(4-(3-chlorophenyl)-4-oxo-1-phenylbutyl)acetamide (6.25 g, 19.79 mmol), 40 mL 6*M* Hydrochloric acid aqueous solution and 120 ml e thanol were added sequentially. The reaction mixture was heated to reflux and reacted for 20 h. After separation through silica gel column chromatography (fluent: ethyl acetate/petroleum ether=1/20), The title product compound was gained as a pale yellow solid (3.50 g, 69%) and recrystallised from methylene chloride to yield colourless blocks of (I).

Anal. Calcd for C_16_H_14_Cl_1_N_1_: C, 75.14; H, 5.52; Cl, 13.86; N, 5.48. Found: C, 75.22; H, 5.50; N, 5.45. ^1^H NMR(CDCl3): 1.94(m,1*H*, N—CH—CH_1_),2.83 (m,1*H*, N—CH—CH_1_), 3.02(m, 1H, N=C—CH_1_), 3.15 (m, 1H, N=C—CH_1_), 5.33 (t, 1H,*C*=N—CH_1_),7.28–7.88(m, 9H, Ph—H).

### Refinement

Although all H atoms were visible in difference maps, they were finally placed in geometrically calculated positions, with C—H distances in the range 0.93–0.98 Å, and included in the final refinement in the riding model approximation,with *U*_iso_(H) = 1.1*U*_eq_(C, N) and *U*_iso_(H) = 1.1*U*_eq_(C).

### Crystal data

**Table:**

C_16_H_14_ClN	*F*(000) = 536
*M**_r_* = 255.73	*D*_x_ = 1.278 Mg m^−^^3^
Monoclinic, *P*2_1_/*c*	Mo *K*α radiation, λ = 0.71073 Å
Hall symbol: -P 2ybc	Cell parameters from 2445 reflections
*a* = 18.2543 (18) Å	θ = 3.2–25.0°
*b* = 5.6398 (5) Å	µ = 0.27 mm^−^^1^
*c* = 13.0095 (13) Å	*T* = 296 K
β = 97.129 (2)°	Block, colourless
*V* = 1329.0 (2) Å^3^	0.38 × 0.32 × 0.20 mm
*Z* = 4	

### Data collection

**Table:**

Bruker SMART CCD diffractometer	2344 independent reflections
Radiation source: fine-focus sealed tube	1856 reflections with *I* > 2σ(*I*)
graphite	*R*_int_ = 0.017
phi and ω scans	θ_max_ = 25.0°, θ_min_ = 3.2°
Absorption correction: multi-scan (*SADABS*; Bruker, 2001)	*h* = −21→21
*T*_min_ = 0.905, *T*_max_ = 0.948	*k* = −6→4
6409 measured reflections	*l* = −14→15

### Refinement

**Table:**

Refinement on *F*^2^	Primary atom site location: structure-invariant direct methods
Least-squares matrix: full	Secondary atom site location: difference Fourier map
*R*[*F*^2^ > 2σ(*F*^2^)] = 0.033	Hydrogen site location: inferred from neighbouring sites
*wR*(*F*^2^) = 0.091	H-atom parameters constrained
*S* = 1.06	*w* = 1/[σ^2^(*F*_o_^2^) + (0.0407*P*)^2^ + 0.239*P*] where *P* = (*F*_o_^2^ + 2*F*_c_^2^)/3
2344 reflections	(Δ/σ)_max_ < 0.001
163 parameters	Δρ_max_ = 0.13 e Å^−^^3^
0 restraints	Δρ_min_ = −0.18 e Å^−^^3^

### Special details

**Table:**

Geometry. All e.s.d.'s (except the e.s.d. in the dihedral angle between two l.s. planes) are estimated using the full covariance matrix. The cell e.s.d.'s are taken into account individually in the estimation of e.s.d.'s in distances, angles and torsion angles; correlations between e.s.d.'s in cell parameters are only used when they are defined by crystal symmetry. An approximate (isotropic) treatment of cell e.s.d.'s is used for estimating e.s.d.'s involving l.s. planes.

### Fractional atomic coordinates and isotropic or equivalent isotropic displacement parameters (Å^2^)

**Table:**

	*x*	*y*	*z*	*U*_iso_*/*U*_eq_	
Cl1	0.46600 (3)	−0.37904 (9)	0.67127 (4)	0.07239 (19)	
N1	0.26607 (7)	0.2985 (3)	0.70282 (10)	0.0606 (4)	
C1	0.36885 (8)	−0.0215 (3)	0.63059 (11)	0.0477 (4)	
H1	0.3644	−0.0242	0.7010	0.057*	
C2	0.41540 (8)	−0.1765 (3)	0.59008 (12)	0.0514 (4)	
C3	0.42310 (9)	−0.1765 (4)	0.48568 (13)	0.0625 (5)	
H3	0.4547	−0.2834	0.4591	0.075*	
C4	0.38333 (9)	−0.0163 (4)	0.42214 (13)	0.0669 (5)	
H4	0.3883	−0.0140	0.3518	0.080*	
C5	0.33616 (9)	0.1414 (3)	0.46108 (12)	0.0573 (4)	
H5	0.3096	0.2492	0.4170	0.069*	
C6	0.32813 (8)	0.1401 (3)	0.56578 (11)	0.0462 (4)	
C7	0.27682 (8)	0.3056 (3)	0.60801 (11)	0.0480 (4)	
C8	0.23478 (9)	0.4944 (3)	0.54365 (13)	0.0548 (4)	
H8A	0.2677	0.6161	0.5233	0.066*	
H8B	0.2070	0.4274	0.4820	0.066*	
C9	0.18419 (12)	0.5915 (4)	0.61625 (14)	0.0760 (6)	
H9A	0.1337	0.5408	0.5957	0.091*	
H9B	0.1857	0.7635	0.6174	0.091*	
C10	0.21413 (9)	0.4885 (3)	0.72331 (13)	0.0589 (4)	
H10	0.2420	0.6131	0.7636	0.071*	
C11	0.15575 (8)	0.3978 (3)	0.78499 (12)	0.0505 (4)	
C12	0.13832 (10)	0.5176 (3)	0.87106 (13)	0.0601 (5)	
H12	0.1635	0.6565	0.8916	0.072*	
C13	0.08427 (11)	0.4353 (4)	0.92721 (16)	0.0761 (6)	
H13	0.0735	0.5184	0.9852	0.091*	
C14	0.04680 (11)	0.2338 (4)	0.89818 (18)	0.0795 (6)	
H14	0.0102	0.1793	0.9360	0.095*	
C15	0.06289 (12)	0.1112 (4)	0.8137 (2)	0.0839 (6)	
H15	0.0373	−0.0272	0.7937	0.101*	
C16	0.11725 (11)	0.1926 (4)	0.75748 (16)	0.0719 (5)	
H16	0.1281	0.1073	0.7001	0.086*	

### Atomic displacement parameters (Å^2^)

**Table:**

	*U*^11^	*U*^22^	*U*^33^	*U*^12^	*U*^13^	*U*^23^
Cl1	0.0734 (3)	0.0730 (3)	0.0733 (3)	0.0237 (2)	0.0194 (2)	0.0061 (2)
N1	0.0599 (8)	0.0775 (10)	0.0437 (8)	0.0241 (8)	0.0038 (6)	−0.0048 (7)
C1	0.0469 (8)	0.0571 (10)	0.0401 (8)	−0.0006 (7)	0.0087 (6)	−0.0050 (7)
C2	0.0442 (8)	0.0575 (10)	0.0536 (9)	−0.0003 (7)	0.0105 (7)	−0.0047 (8)
C3	0.0532 (9)	0.0790 (13)	0.0581 (10)	0.0053 (9)	0.0177 (8)	−0.0161 (10)
C4	0.0605 (10)	0.1011 (15)	0.0413 (9)	0.0014 (10)	0.0155 (8)	−0.0081 (10)
C5	0.0534 (9)	0.0760 (12)	0.0425 (9)	0.0009 (9)	0.0064 (7)	0.0010 (8)
C6	0.0426 (8)	0.0557 (10)	0.0402 (8)	−0.0028 (7)	0.0046 (6)	−0.0057 (7)
C7	0.0467 (8)	0.0550 (10)	0.0415 (8)	0.0014 (7)	0.0019 (6)	−0.0047 (7)
C8	0.0580 (9)	0.0512 (10)	0.0542 (9)	−0.0003 (8)	0.0031 (8)	0.0030 (8)
C9	0.0848 (13)	0.0769 (14)	0.0667 (12)	0.0310 (11)	0.0118 (10)	0.0077 (10)
C10	0.0606 (10)	0.0617 (11)	0.0543 (10)	0.0154 (9)	0.0061 (8)	−0.0110 (9)
C11	0.0526 (9)	0.0463 (9)	0.0503 (9)	0.0123 (7)	−0.0026 (7)	−0.0103 (7)
C12	0.0645 (10)	0.0562 (11)	0.0597 (10)	0.0026 (9)	0.0080 (8)	−0.0168 (9)
C13	0.0760 (13)	0.0863 (15)	0.0690 (12)	0.0065 (11)	0.0213 (10)	−0.0087 (11)
C14	0.0666 (12)	0.0777 (15)	0.0946 (16)	0.0033 (11)	0.0111 (11)	0.0177 (13)
C15	0.0753 (13)	0.0512 (12)	0.1202 (19)	−0.0043 (10)	−0.0082 (13)	−0.0044 (12)
C16	0.0749 (12)	0.0584 (12)	0.0803 (13)	0.0084 (10)	0.0011 (10)	−0.0247 (10)

### Geometric parameters (Å, °)

**Table:**

Cl1—C2	1.7410 (17)	C8—H8B	0.9700
N1—C7	1.2733 (19)	C9—C10	1.545 (2)
N1—C10	1.477 (2)	C9—H9A	0.9700
C1—C2	1.369 (2)	C9—H9B	0.9700
C1—C6	1.392 (2)	C10—C11	1.501 (2)
C1—H1	0.9300	C10—H10	0.9800
C2—C3	1.383 (2)	C11—C16	1.378 (2)
C3—C4	1.370 (3)	C11—C12	1.378 (2)
C3—H3	0.9300	C12—C13	1.379 (3)
C4—C5	1.377 (2)	C12—H12	0.9300
C4—H4	0.9300	C13—C14	1.356 (3)
C5—C6	1.388 (2)	C13—H13	0.9300
C5—H5	0.9300	C14—C15	1.361 (3)
C6—C7	1.476 (2)	C14—H14	0.9300
C7—C8	1.505 (2)	C15—C16	1.382 (3)
C8—C9	1.504 (2)	C15—H15	0.9300
C8—H8A	0.9700	C16—H16	0.9300
			
C7—N1—C10	109.37 (14)	C8—C9—H9A	110.8
C2—C1—C6	119.66 (14)	C10—C9—H9A	110.8
C2—C1—H1	120.2	C8—C9—H9B	110.8
C6—C1—H1	120.2	C10—C9—H9B	110.8
C1—C2—C3	121.45 (16)	H9A—C9—H9B	108.9
C1—C2—Cl1	119.55 (12)	N1—C10—C11	111.31 (15)
C3—C2—Cl1	119.01 (13)	N1—C10—C9	105.86 (13)
C4—C3—C2	118.80 (16)	C11—C10—C9	114.51 (14)
C4—C3—H3	120.6	N1—C10—H10	108.3
C2—C3—H3	120.6	C11—C10—H10	108.3
C3—C4—C5	120.82 (15)	C9—C10—H10	108.3
C3—C4—H4	119.6	C16—C11—C12	117.45 (17)
C5—C4—H4	119.6	C16—C11—C10	121.35 (15)
C4—C5—C6	120.29 (16)	C12—C11—C10	121.19 (16)
C4—C5—H5	119.9	C11—C12—C13	121.18 (18)
C6—C5—H5	119.9	C11—C12—H12	119.4
C5—C6—C1	118.98 (15)	C13—C12—H12	119.4
C5—C6—C7	120.74 (15)	C14—C13—C12	120.27 (19)
C1—C6—C7	120.28 (13)	C14—C13—H13	119.9
N1—C7—C6	121.47 (14)	C12—C13—H13	119.9
N1—C7—C8	115.58 (14)	C13—C14—C15	119.9 (2)
C6—C7—C8	122.94 (13)	C13—C14—H14	120.0
C9—C8—C7	102.64 (14)	C15—C14—H14	120.0
C9—C8—H8A	111.2	C14—C15—C16	120.0 (2)
C7—C8—H8A	111.2	C14—C15—H15	120.0
C9—C8—H8B	111.2	C16—C15—H15	120.0
C7—C8—H8B	111.2	C11—C16—C15	121.21 (18)
H8A—C8—H8B	109.2	C11—C16—H16	119.4
C8—C9—C10	104.67 (14)	C15—C16—H16	119.4
			
C6—C1—C2—C3	0.0 (2)	C7—C8—C9—C10	12.4 (2)
C6—C1—C2—Cl1	−179.78 (12)	C7—N1—C10—C11	134.21 (15)
C1—C2—C3—C4	0.4 (3)	C7—N1—C10—C9	9.2 (2)
Cl1—C2—C3—C4	−179.80 (14)	C8—C9—C10—N1	−13.5 (2)
C2—C3—C4—C5	−0.4 (3)	C8—C9—C10—C11	−136.50 (16)
C3—C4—C5—C6	−0.1 (3)	N1—C10—C11—C16	−48.5 (2)
C4—C5—C6—C1	0.5 (2)	C9—C10—C11—C16	71.5 (2)
C4—C5—C6—C7	−178.99 (16)	N1—C10—C11—C12	131.90 (16)
C2—C1—C6—C5	−0.5 (2)	C9—C10—C11—C12	−108.09 (19)
C2—C1—C6—C7	179.01 (14)	C16—C11—C12—C13	−0.2 (3)
C10—N1—C7—C6	178.25 (14)	C10—C11—C12—C13	179.46 (17)
C10—N1—C7—C8	−1.0 (2)	C11—C12—C13—C14	−0.3 (3)
C5—C6—C7—N1	176.41 (16)	C12—C13—C14—C15	0.4 (3)
C1—C6—C7—N1	−3.0 (2)	C13—C14—C15—C16	−0.1 (3)
C5—C6—C7—C8	−4.4 (2)	C12—C11—C16—C15	0.5 (3)
C1—C6—C7—C8	176.10 (14)	C10—C11—C16—C15	−179.16 (17)
N1—C7—C8—C9	−7.8 (2)	C14—C15—C16—C11	−0.3 (3)
C6—C7—C8—C9	173.00 (15)		
